# The complete chloroplast genome of *Mentha spicata,* an endangered species native to South Europe

**DOI:** 10.1080/23802359.2017.1413311

**Published:** 2017-12-07

**Authors:** Kangyu Wang, Li Li, Ying Hua, Mingzhu Zhao, Shaokun Li, Honghua Sun, Yanxi Lv, Yi Wang

**Affiliations:** a College of Life Science, Jilin Agricultural University, Changchun, China;; b Jilin ZhongKe Bio-engineering Joint Stock Co., Ltd, Changchun, China

**Keywords:** *Mentha spicata*, chloroplast genome, HomBlocks, phylogenomics, phylogenetic relationship

## Abstract

*Mentha spicata,* also known as mint, is the best known source of aromatic essential oil. It was widespread in Europe and Asia. But due to human activity, it has been classified as Least Concern (LC) species in the IUCN Red List of Threatened Species. Here, we presented the complete chloroplast genome of *M. spicata*. The circular genome is 16,430 bp in length and contains 132 genes, including 87 protein-coding genes (PCG), 37 transfer RNA genes (tRNA) and eight ribosomal RNA genes (rRNA). The overall nucleotide composition is: 30.7% A, 19.2% C, 18.6% G, 31.5% T, with a total G + C content of 37.85%. The phylogenetic tree was constructed to explore the taxonomic status of *M. spicata*, which contributes to phylogenetic studies and further conservation strategies for this species.

*Mentha spicata,* also known as mint, belongs to a genus of plants in the family Lamiaceae. For decades, *M. spicata* is used to extract aromatic essential oil, which is the source of the best-known monoterpenes, menthol and carvone (Jin et al. 2014). They are extensively used in flavour and fragrance industries, pharmaceuticals and cosmetic products (Lange et al. 2011). *M. spicata* has a very widespread distribution, but due to its long-term and excessive utilization by man, the amount of this species has significantly declined. In 2014, they were classified as Least Concern(LC) species in the IUCN Red List of Threatened Species (Lansdown 2014). The availability of the complete chloroplast genome sequences is helpful to the conservation of endangered species. However, sequences of complete chloroplast genomes of the genus *Mentha* is limited to *M. longifolia* (Vining et al. 2017). In this study, we obtained and characterized the complete chloroplast genome sequence of *M. spicata* using the Illumina paired-end sequencing data, which will contribute to develop protection measures for this endangered species.

The specimen of *M. spicata* was isolated from Jilin Agricultural University test field in Changchun, Jilin, China (125.24E; 43.48N) and the DNA of *M. spicata* was stored in Jilin Agricultural University College of Life Science (No. JLAUCLS3). The DNA sample was sequenced using the Illumina X-Ten Sequencing Platform (Illumina, CA). Before chloroplast genome assembling, adapters and low-quality sequences were removed using the FastQC software (Andrews 2010). The chloroplast genome was assembled with SPAdes v3.8 (http://bioinf.spbau.ru/spades) (Bankevich et al. 2012) and annotated by DOGMA (http://dogma.ccbb.utexas.edu/) (Wyman et al. 2004). The tRNA genes were further identified using ARAGORN (Laslett and Canback 2004). The annotated chloroplast genome was submitted to GenBank database under accession No. MG256495.

The complete chloroplast genome of *M. spicata* is a circular molecule with a length of 152,132bp, comprising a pair of inverted repeat regions (IRs) of 25,625bp, a large single-copy region (LSC) of 83,218bp and a small single-copy region (SSC) of 17,664bp. It contained 132 genes, including 87 protein-coding genes (PCG), 37 transfer RNA genes (tRNA) and eight ribosomal RNA genes (rRNA). Among those genes, 17 genes duplicated in the IR region. Additionally, 20 genes were found containing a single intron and two genes (*ClpP & ycf3*) had two introns. The base compositions of *M. spicata* chloroplast genome were uneven (30.7% A, 19.2% C, 18.6% G, 31.5% T). The overall GC content of this chloroplast genome was 37.85%.

**Figure 1.**
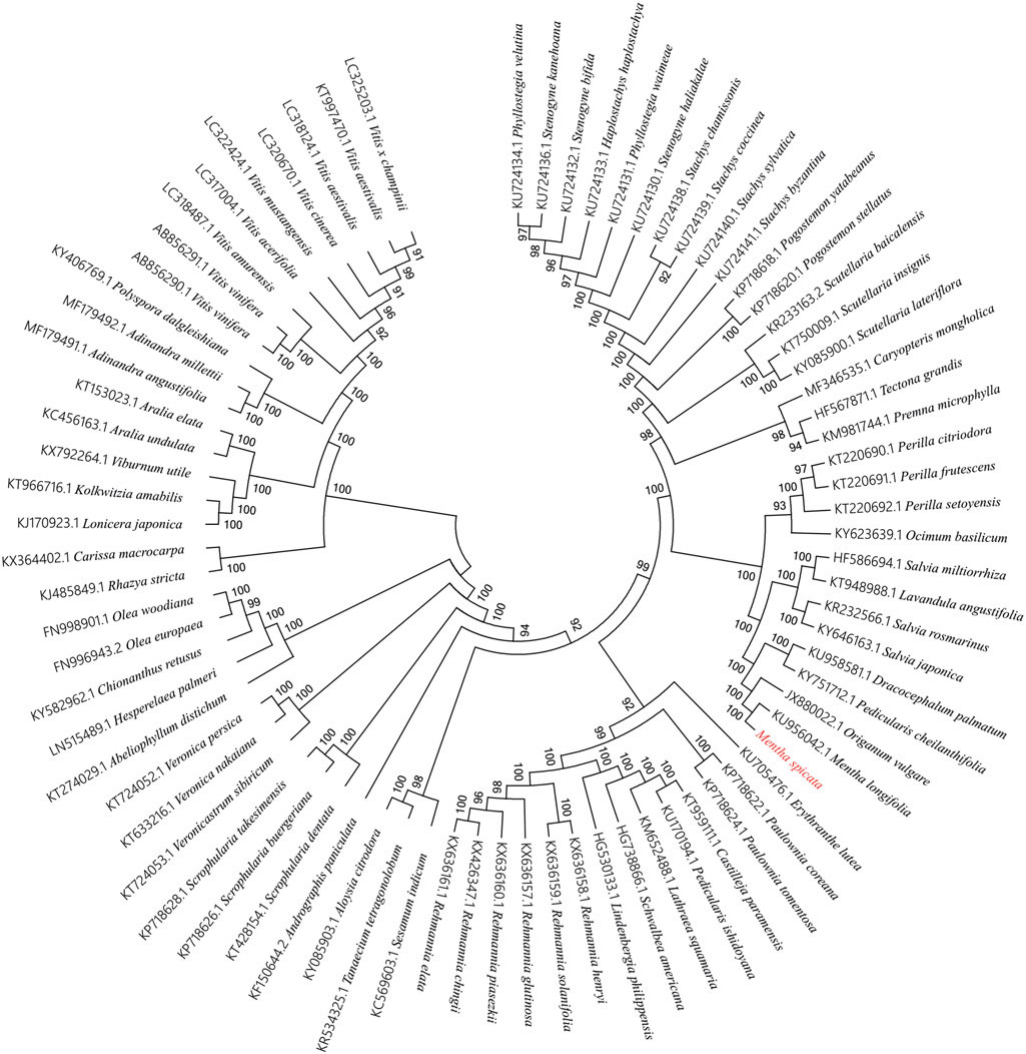
Phylogenetic relationships among 79 plant chloroplast genomes. This tree was drawn without setting of an outgroup. All nodes exhibit above 90% bootstraps. The length of branch represents the divergence distance.

To ascertain phylogenetic position of *M. spicata* among other higher plants, we selected 79 published complete chloroplast genome sequences of higher plants to construct alignment using Homblocks (https://github.com/fenghen360/HomBlocks) (Bi et al. 2017). The phylogenetic trees were reconstructed using maximum-likelihood (ML) and neighbour-joining (NJ) methods. ML analysis were performed using RaxML-8.2.4 (Stamatakis 2014), of which the bootstrap values were calculated using 1000 replicates to assess node support. NJ phylogenetic tree was constructed using MEGA7 with 1000 bootstrap replicate (Kumar et al. 2016). All the nodes were inferred with strong support by the ML and NJ methods. As shown in the phylogenetic tree (Figure 1), The chloroplast genome of *M. spicata* was clustered with *M. longifolia* and *Origanum vulgare.*
